# Supplementary material for: A comparative analysis of HIV status by sociodemographic and sexual behavior characteristics among men in Bexar county, Texas: An Ending the HIV Epidemic priority county
Source: PLoS One. 2026 Mar 2;21(3):e0333665. doi: 10.1371/journal.pone.0333665 (PMC12952590; doi:10.1371/journal.pone.0333665)
Supplement: S2 File — (PDF) [file pone.0333665.s002.pdf]

File Information

Notes

Output Created		13-FEB-2026 09:04:24
Comments		
Input	Data	C:\Users\mix498\Downloads\S1 (1).sav
	Active Dataset	DataSet1
	Filter	<none>
	Weight	<none>
	Split File	<none>
Syntax		DISPLAY DICTIONARY.
Resources	Processor Time	00:00:00.00
	Elapsed Time	00:00:00.02

```
[DataSet1] C:\Users\mix498\Downloads\S1 (1).sav
```

Variable Information

Variable	Position	Label	Measurement Level	Role	Column Width
Knowledge.of.Status	1	Knows HIV status (screening item)	Nominal	Input	15
Current.Status	2	Current HIV status among those who know their status	Nominal	Input	12
Current.Status.Unknown	3	Self-reported HIV status (derived 3-category)	Nominal	Input	8
Race.Ethn	4	Race/ethnicity (3-category)	Nominal	Input	10
Race.Eth.Binary	5	BIPOC indicator (Hispanic/Latino or Black vs White)	Nominal	Input	10

Variable Information

Variable	Alignment	Print Format	Write Format
Knowledge.of.Status	Right	F8.2	F8.2
Current.Status	Right	F8.2	F8.2
Current.Status.Unknown	Right	F8.2	F8.2
Race.Ethn	Right	F8.2	F8.2
Race.Eth.Binary	Right	F8.2	F8.2

Variable Information

Variable	Position	Label	Measurement Level	Role	Column Width
Education	6	Highest education (6-category; ordered)	Ordinal	Input	11
Income	7	Household income (12-category; ordered)	Ordinal	Input	10
Sex	8	Reported gender identification	Nominal	Input	10
Employment	9	Employment status	Nominal	Input	12
Spouses	10	Marital status	Nominal	Input	10
MentalHealth	11	Mental health category (3-level coded)	Nominal	Input	14
DrugMisuse	12	Drug misuse indicator	Nominal	Input	12
Treatment	13	Currently/ever in treatment	Nominal	Input	11
SexuallyActive	14	Sexually active in last 30 days	Nominal	Input	16
CasuallySex	15	Casual sex ('hooked up') in last 6 months	Nominal	Input	13
AnalSex	16	Ever had anal sex	Nominal	Input	10
STIs	17	History of STI (binary)	Nominal	Input	10
Condoms	18	Condom use frequency (ordered	Ordinal	Input	10
Drugs	19	Drug/substance use during sex frequency (ordered)	Ordinal	Input	10
Alcohol	20	Alcohol intoxication during sex frequency (ordered)	Ordinal	Input	10

Variable Information

Variable	Alignment	Print Format	Write Format
Education	Right	F8.2	F8.2
Income	Right	F8.2	F8.2
Sex	Right	F8.2	F8.2
Employment	Right	F8.2	F8.2
Spouses	Right	F8.2	F8.2
MentalHealth	Right	F8.2	F8.2
DrugMisuse	Right	F8.2	F8.2
Treatment	Right	F8.2	F8.2
SexuallyActive	Right	F8.2	F8.2
CasuallySex	Right	F8.2	F8.2
AnalSex	Right	F8.2	F8.2
STIs	Right	F8.2	F8.2
Condoms	Right	F8.2	F8.2
Drugs	Right	F8.2	F8.2
Alcohol	Right	F8.2	F8.2

Variable Information

Variable	Position	Label	Measurement Level	Role	Column Width
SexualOrientation	21	Self reported sexual orientation	Nominal	Input	8
MSM	22	Self reported MSM	Nominal	Input	8

Variable Information

Variable	Alignment	Print Format	Write Format
SexualOrientation	Right	F8.2	F8.2
MSM	Right	F8.2	F8.2

Variables in the working file

Variable Values

Value		Label
Knowledge.of.Status	.00	No
	1.00	Yes
Current.Status	.00	HIV Negative
	1.00	HIV Positive
Current.Status.Unknown	.00	HIV Negative
	1.00	HIV Positive
	2.00	Does not know HIV status
Race.Ethn	.00	Hispanic/Latino
	1.00	NH Black
	2.00	NH White
Race.Eth.Binary	.00	Person of color
	1.00	NH White
Education	.00	Less than high school
	1.00	High school graduate
	2.00	Technical degree
	3.00	Some college
	4.00	Undergraduate degree
	5.00	Graduate degree
Income	.00	Less than $10,000
	1.00	$10,000 - $19,999
	2.00	$20,000 - $29,999

Variable Values

Value		Label
	3.00	$30,000 - $39,999
	4.00	$40,000 - $49,999
	5.00	$50,000 - $59,999
	6.00	$60,000 - $69,999
	7.00	$70,000 - $79,999
	8.00	$80,000 - $89,999
	9.00	$90,000 - $99,999
	10.00	$100,000 - $149,999
	11.00	More than $150,000
Sex	.00	Female
	1.00	Male
Employment	.00	Unemployed
	1.00	Employed
Spouses	.00	Single
	1.00	Married
MentalHealth	.00	I have not been diagnosed with any mental health disorders
	1.00	Anxiety
	2.00	Depression
DrugMisuse	.00	I have not been diagnosed with any drug and alcohol use disorders
	1.00	Drug/Substance Misuse/Alcoholism
Treatment	.00	I have not been diagnosed with any mental health or drug and alcohol use disorders
	1.00	Currently in treatment
SexuallyActive	.00	No
	1.00	Yes
CasuallySex	.00	No
	1.00	Yes
AnalSex	.00	No
	1.00	Yes
STIs	.00	No
	1.00	Yes
Condoms	.00	Never
	1.00	Sometimes

Variable Values

Value		Label
	2.00	About half the time
	3.00	Most of the time
	4.00	Always
Drugs	.00	Never
	1.00	Sometimes
	2.00	About half the time
	3.00	Most of the time
	4.00	Always
Alcohol	.00	Never
	1.00	Sometimes
	2.00	About half the time
	3.00	Most of the time
	4.00	Always
SexualOrientation	.00	Straight
	1.00	Bisexual
	2.00	Gay
MSM	.00	Not MSM
	1.00	MSM
