# Supplementary material for: A comparative analysis of HIV status by sociodemographic and sexual behavior characteristics among men in Bexar county, Texas: An Ending the HIV Epidemic priority county
Source: PLoS One. 2026 Mar 2;21(3):e0333665. doi: 10.1371/journal.pone.0333665 (PMC12952590; doi:10.1371/journal.pone.0333665)
Supplement: Attachment — Submitted filename: Response to reviewers.docx [file pone.0333665.s004.docx]

# **Response to Reviewers**

### Manuscript title: *A Comparative Analysis of HIV Status by Sociodemographic and Sexual Behavior Characteristics among Men in Bexar County, Texas: An Ending the HIV Epidemic (EHE) Targeted County* Journal: PLOS ONE Manuscript ID: *PONE-D-25-10308*

### **Overview of Revisions**

We thank the editor and reviewer for their thoughtful and constructive feedback. We have revised the manuscript accordingly and respond point-by-point to each comment below. Major revisions include: (1) an expanded discussion of sample representativeness; (2) clarification of eligibility criteria and justification for the men-only analytic sample; (3) addition of a Data Quality Assurance subsection; (4) substantial condensation and reorganization of the Discussion; (5) clearer articulation of the study’s main contributions; and (6) updated Competing Interests, Ethics, and Data Availability statements to ensure full compliance with PLOS ONE requirements. Detailed responses are provided below, with revised manuscript text quoted where appropriate..

## Response to Reviewer #1

## Comment 1: Unfortunately the sample disproportionately non-Hispanic White (63%) compared to the actual demographic profile of Bexar County (majority Latino) and calls to question the representativeness of the findings for Bexar County.

Response: We agree that the racial/ethnic composition of our sample limits its representativeness for Bexar County. In the Limitations section, we now explicitly acknowledge that the sample consists primarily of non-Hispanic White men, whereas Bexar County is majority Latino, describe how our recruitment approach likely contributed to this imbalance, and clarify that the findings should be interpreted as describing a convenience sample of men engaged with or reachable through these channels rather than all men in the county. We also note that future work should employ targeted recruitment or oversampling to ensure adequate representation of Latino and other underrepresented groups. At the same time, we believe the behavioral patterns observed in this service-engaged, locally recruited sample remain informative for Bexar County’s Ending the HIV Epidemic efforts, because they characterize the sexual and substance-use behaviors of men who are currently connected to, or reachable through, local HIV prevention and care networks. We clarify in the revised manuscript that our conclusions are framed in terms of these behavioral profiles within this subset of men, rather than as population-representative estimates for all men in Bexar County.

## Comment 2: The conclusion that HIV-negative men engage in more high-risk behaviors yet remain HIV-negative due to education/economic stability is speculative… Greater caution is needed in these interpretations.

Response: We appreciate this comment and agree that our original wording overstated the interpretation of these findings. In response, we revised the final sentences of the Abstract to describe patterns of HIV status awareness as cross-sectional associations between sociodemographic and behavioral characteristics, rather than as evidence that education or economic stability explain why some men remain HIV-negative. The revised abstract now emphasizes that men who know their HIV status, particularly those who are HIV-negative, more often occupy socioeconomically advantaged positions and report distinct sexual behavior profiles compared with men whose status is unknown, and that these associations should be interpreted cautiously. We also revised the Discussion to highlight the limitations of our cross-sectional, self-reported data and to note that biological and temporal factors, such as recent exposure, the HIV testing window period, and differential access to testing, may also contribute to the observed patterns. We now explicitly frame the results as hypothesis-generating rather than as definitive evidence of causal pathways. We also revised the Results section to ensure that all descriptions of the multinomial logistic regression models correctly reflect the reference categories and direction of the odds ratios and avoid implying causal explanations.

## Comment 3: The study limits the analytic sample to men, but no clear justification is provided for this decision. While men who have sex with men are indeed a key population for HIV prevention, excluding women and gender-diverse individuals without explanation diminishes the broader relevance of the study and risks overlooking disparities in other groups in Bexar County.

Response: We have revised the Materials and methods to clarify that the analytic sample was restricted to men as a methodological decision grounded in the behavioral focus of the survey instrument, which was developed to capture sexual and substance-use behaviors particularly salient for HIV risk and status awareness among men in this local context. The survey content and recruitment strategy were designed in collaboration with community partners whose prevention and testing programs predominantly serve men at elevated HIV risk, and the analytic restriction aligns the sample with the population for whom these data were collected and the interventions are intended.

## Comment 4: The description of eligibility is unclear. The manuscript states that participants were eligible if they consented, were ≥18 years old, and “indicated their HIV status.” However, it is not defined what “indicating” HIV status entails.

Response: We agree that the phrase “indicated their HIV status” was unclear. We have revised the Materials and methods (Study Design and Data Collection) to specify that, after providing electronic consent, participants were asked two HIV status awareness items: “Do you know your HIV status?” and, for those who answered “Yes,” “What is your current HIV status?” Eligibility required responding to the first item and, for participants who reported knowing their status, the second item. In the Measures section, we further describe this two-step process in detail and clarify that we used these responses to construct a three-category HIV status variable (HIV-negative, HIV-positive, unknown HIV status) based entirely on self-report.

## Comment 5: As the study relied on an online Qualtrics survey distributed via social media and email, procedures to detect and prevent fraudulent responses (e.g., bots, duplicate entries, ineligible participants) should be clearly described. The manuscript does not indicate whether tools such as CAPTCHA, IP address checks, or survey completion time thresholds were used. This omission raises concerns about data quality and the validity of the analytic sample.

Response: We appreciate this comment and agree that our procedures for assessing data quality in the online survey required clearer description. In the Materials and methods (Study Design and Data Collection), we now describe the steps we took to screen for implausible or potentially fraudulent responses. Specifically, we report that the Qualtrics platform recorded completion time and response patterns for each case and that we examined these metadata for implausibly short completion times, highly patterned response sets, and obviously duplicated entries based on key demographic variables and HIV status. No responses met our criteria for exclusion, and all eligible cases were retained. We did not implement additional automated tools such as CAPTCHA or IP-based blocking, and we now note in the Limitations that, despite these checks, the possibility of undetected fraudulent or duplicate responses cannot be entirely ruled out, which is a general limitation of anonymous online survey research.

## Comment 6: The Discussion is lengthy and at times disorganized, with substantial repetition of results. The authors should reduce the regurgitation of descriptive findings and instead focus on the key implications for HIV prevention and public health policy.

Response: We substantially revised the Discussion to reduce repetition of descriptive findings and improve organization. Specifically, we removed detailed restatements of percentages and table values and replaced them with a more concise summary of the main sociodemographic and behavioral patterns across HIV-negative, HIV-positive, and unknown-status men. We then refocused the Discussion on key implications for HIV prevention and EHE implementation in Bexar County, including the profiles of men with unknown status, the role of socioeconomic position and service engagement in shaping status awareness, and how these patterns can inform targeted testing and outreach strategies. We also clarified that the results are hypothesis-generating, given the cross-sectional design and self-reported measures, rather than definitive evidence of causal pathways.

## Comment 7: Many of the associations (education, employment, condom use, income) are already well-established in the HIV literature. The true contributions of this study are: providing county-specific data in an EHE target area, and highlighting the characteristics of individuals with “unknown HIV status.”The manuscript should make this clear, rather than suggesting the findings themselves are novel.

Response: We agree that many of the sociodemographic and behavioral associations we observed (e.g., with education, employment, income, and condom use) are well established in the HIV literature, and that the primary contribution of this study lies in its local focus and characterization of men with unknown HIV status. In the revised Discussion, we removed language that could be interpreted as suggesting that the associations themselves are novel. Instead, in the concluding paragraph, we now state explicitly that the contribution of this work is to provide county-specific evidence from an EHE target area and to describe men with unknown HIV status as a distinct and under-reached group within a local, service-engaged male population. We also clarify that the findings are hypothesis-generating and are intended to inform locally tailored testing and outreach strategies, rather than to claim discovery of new risk or protective factors.

## **Response to Journal Requirements**

## 1. **PLOS ONE style and formatting**

Response: We have revised the manuscript to conform to PLOS ONE’s formatting requirements and file-naming conventions, following the templates provided at the links in your letter. In particular, we have ensured that section headings, title page information, tables, and figure labels match the PLOS ONE style.

## 2. **Competing Interests statement**

Response: Our updated Competing Interests statement is as follows: Adolph J. Delgado has an ongoing consulting relationship with the Alamo Area Resource Center (AARC) for grant development, advising, and program evaluation services. This consulting arrangement is independent of the present study and did not influence the study design, data collection, analysis, interpretation, or decision to publish. The other authors declare that they have no competing interests. This does not alter our adherence to PLOS ONE policies on sharing data and materials.

**Ethics Statement placement**

Response: We have ensured that the ethics statement appears only in the Methods section of the manuscript and have removed it from all other sections.

## 3. **Data Availability and data sharing**

Response: There are no legal or ethical restrictions that prevent us from sharing a de-identified version of the dataset used in this study. In accordance with PLOS ONE’s data policy, we have prepared the minimal anonymized dataset necessary to replicate our findings and are making it publicly available.

## 4. **Reviewer-suggested citations**

Author Response: No new citations were required by the reviewer.
